# Serendipitous compound action potential oscillations reveal glycolytic astrocyte and oxidative axon interstitial K^+^ buffering in central white matter

**DOI:** 10.1113/EP093107

**Published:** 2025-11-10

**Authors:** Amy J. Hopper, Angus M. Brown

**Affiliations:** ^1^ School of Life Sciences University of Nottingham Nottingham UK; ^2^ Department of Neurology University of Washington Seattle Washington USA

**Keywords:** astrocyte, axon, buffering, compound action potential, glycolysis, lactate, mouse optic nerve

## Abstract

The principal processes that govern interstitial K^+^ ([K^+^]_o_) buffering in mouse optic nerve (MON), a central white matter tract, either directly consume energy (Na^+^–K^+^‐ATPase) or use transmembrane ion gradients created by energy‐dependent pumps to enable the K^+^ fluxes that maintain a stable [K^+^]_o_, and thus ready availability of utilisable energy substrate is vital in supporting MON function. We switched the artificial cerebrospinal fluid (aCSF) bathing isolated *ex vivo* MON from a glucose and physiological [K^+^] (3 mM) formulation to one in which glucose was replaced by lactate and [K^+^] was increased to supra‐physiological concentrations (‘stress aCSF’), to test the ability of an oxidative fuel to support astrocyte function when faced with the buffering‐related increased energy demand that accompanies elevating [K^+^]_o_. We recorded simultaneously the compound action potential (CAP) and [K^+^]_o_ with suction electrodes and ion‐sensitive microelectrodes, respectively. Increases in aCSF [K^+^] were not matched by equivalent increases in [K^+^]_o_, evidence of powerful buffering. The stress aCSF caused unexpected reciprocal CAP and [K^+^]_o_ oscillations and exhaustion of astrocyte energy reserves coupled with elevation of [K^+^]_o_ sufficient to activate axonal Na^+^ channels, the key factors required for their initiation. The oscillation profile was of a rise in [K^+^]_o_ towards aCSF [K^+^], followed by a restoration of [K^+^]_o_ towards baseline, driven by intermittent activation of the axonal Na^+^–K^+^‐ATPase, a cyclical process that continued for several hours. These oscillations exposed the contrasting utility of lactate, supporting axonal CAPs and axonal dominance of buffering during the oscillations, but incapable of fuelling astrocyte function.

## Abstract (highlights)

- **What is the central question of this study?**
  How does the central white matter buffer interstitial K^+^ when placed under metabolic stress?
- **What is the main finding and its importance?**
  At rest, interstitial K^+^ buffering relies on astrocytes, but when bathing K^+^ is raised and lactate is the sole available energy substrate, axonal buffering predominates.

## 1 INTRODUCTION

The brain's interstitial space is a privileged environment, its ionic milieu secreted by the choroid plexus (Ransom, 2009) and meticulously protected from systemic ion fluctuations by the blood–brain barrier (Bradbury & Davson, 1965). Ion‐specific transporters, pumps, channels and exchangers are present on neural cells (Macaulay & Zeuthen, 2012), which respond rapidly to interstitial ion perturbations, maintaining an ionic environment tailored to optimise neuronal conduction (Hille, 2001). However, this view of an ideal homeostatic norm has been challenged on the grounds that evolution has found use for physiological ion fluctuations (Somjen, 1979). This applies particularly to [K^+^]_o_, which rises following neural activity and stimulates lactate release from astrocytes, fuelling neuronal energy demand (Bittner et al., 2011; Looser et al., 2024; Ruminot et al., 2011; Sotelo‐Hitschfeld et al., 2015).

Increasing [K^+^]_o_ depolarises neural membrane potentials (Sawyer et al., 2017). Since activity causes all neurones to release K^+^ to the interstitial fluid (Frankenhaeuser & Hodgkin, 1956), membrane depolarisation inevitably follows activity, which, if unchecked, would lead to a positive feedback cycle of relentless depolarisation (Grafstein, 1956). However, buffering of K^+^ protects the brain from the consequences of unrestricted [K^+^]_o_ elevations (Somjen, 2002): in the mouse optic nerve (MON), activity‐induced increases in [K^+^]_o_ and axonal [Na^+^]_i_ stimulate the Na^+^–K^+^‐ATPase (NKA) on astrocytes and axons, respectively (Ransom et al., 2000), whose combined buffering power is evident in the reduction of [K^+^]_o_ at a rate of 0.25 mM s^−1^ following evoked activity (Hopper et al., 2023).

The pursuit of unravelling the intricacies of [K^+^]_o_ buffering has been ongoing for six decades (Orkand et al., 1966). The activity‐dependent complex interactions amongst various neural cell types, which selectively express discrete K^+^ transport mechanisms, ensure that a consensus view of [K^+^]_o_ buffering remains elusive. The difficulties of studying a system in dynamic equilibrium can be mitigated by studying the way a system recovers after disrupting the equilibrium, a tactic used to successfully investigate pH_i_ regulation (Thomas, 1984). Disrupting [K^+^]_o_ by imposing a high‐frequency stimulus for extended durations is the standard means by which [K^+^]_o_ buffering has been studied for decades (Lothman & Somjen, 1975), the resulting ‘shark fin’ [K^+^]_o_ profile offering a stereotypical response against which experimental interventions can be compared. However, inconsistencies and contradictions abound, for example in the role of Ba^2+^‐sensitive K^+^ flux via Kir channels, where in hippocampus the flux contributes to baseline [K^+^]_o_ and recovery of [K^+^]_o_ post‐stimulus (D'Ambrosio et al., 2002), but does (Bay & Butt, 2012) or does not (Ransom et al., 2000) contribute to post‐stimulus recovery of [K^+^]_o_ levels in rodent optic nerve.

In this paper, we report metabolic stress‐induced [K^+^]_o_ oscillations in MON, with a secondary effect apparent as cyclical loss followed by recovery of the compound action potential (CAP). The oscillations were initiated when astrocyte energy depletion, coupled with an inability to efficiently metabolise oxidative substrates such as lactate, compromised astrocyte buffering capability, leading to a rapid rise in [K^+^]_o_, a unique observation that distinguished these oscillations from previously reported elevations in [K^+^]_o_ of neuronal origin in white matter (Connors & Ransom, 1984) or grey matter (Somjen, 2001, 2002). The oscillations were sustained by intermittent activation of the axonal NKA, stimulated by elevated axonal [Na^+^]_i_, which accumulated on opening of tetrodotoxin (TTX)‐sensitive voltage‐gated Na^+^ channels responding to the axonal membrane depolarisation that is an inevitable consequence of elevated [K^+^]_o_.

## 2 METHODS

### 2.1 Ethical approval

All experiments were approved by the University of Nottingham Animal Care and Ethics Committee and were carried out in accordance with the Animals (Scientific Procedures) Act 1986 under the appropriate authority of establishment (NON ASPA 2321). All investigators understand the ethical principles under which the journal operates and certify that the present study complies with the animal ethics checklist.

### 2.2 Animals

Experiments were performed on female CD‐1 mice (weight 28–35 g, corresponding to 30–45 days of age) purchased from Charles River Laboratories (Margate, UK). Mice were group housed with ad libitum access to food and water and maintained at 22–23°C on a 12:12 h light–dark cycle. Mice were killed by the Schedule 1 method of cervical dislocation; death was confirmed by permanent cessation of circulation. A total of 74 mice were used, providing 111 optic nerve recordings. Each *n* value is treated as a biological unit and constitutes the recording from a perfusion chamber of an individual optic nerve (Rich et al., 2020).

### 2.3 Electrophysiological recordings

The optic nerves were dissected free and cut at the optic chiasm and behind the orbit. The optic nerves were gently freed from their dural sheaths and placed in an interface perfusion chamber (Medical Systems Corp., Greenvale, NY, USA), maintained at 37°C and superfused with control artificial cerebrospinal fluid (aCSF) containing (in mM): NaCl 126, KCl 3.0, CaCl_2_ 2.0, MgCl_2_ 2.0, NaH_2_PO_4_ 1.2, NaHCO_3_ 26 and glucose 10. The chamber was continuously aerated by a humidified gas mixture of 95% O_2_–5% CO_2_. Suction electrodes backfilled with aCSF were used for stimulation and recording (Stys et al., 1991). One electrode was attached to the rostral end of the nerve for stimulation, and the second suction electrode was attached to the caudal end of the nerve to record the CAP; thus, all recordings were orthodromic. A 30 µs supramaximal strength was applied to the optic nerve at 1 Hz unless otherwise stated (Grass S88 Stimulator; Grass Technologies, West Warwick, RI, USA). The signal was amplified 10× by an Axoprobe 1A amplifier (Molecular Devices, San Jose, CA, USA) then amplified a further 100× by a Stanford Research System Preamplifier (SR560, Stanford Research Systems, Sunnyvale, CA, USA), filtered at 30 kHz and acquired at 20 kHz (Digidata 1550B, Molecular Devices) using Clampex 11.0 (Molecular Devices).

### 2.4 K^+^ sensitive microelectrodes

K^+^‐sensitive microelectrodes were made with double‐barrelled piggyback glass (WPI, Sarasota, FL, USA, PB150F‐6) as previously described (Borrelli et al., 1985; Hopper et al., 2023), with slight modifications. Electrodes were broken back to a tip diameter of >5 µm by gentle manual agitation against a 6000‐grit whetstone (Oakes Kitchenware, Ripley, UK). The tip of the ion‐sensitive barrel was back‐filled with *N*,*N*‐dimethyltrimethylsilyamine (41716; Sigma‐Aldrich, Merck Life Science UK Ltd, Gillingham, UK) and baked at 160°C for 1 h (Ambiano mini oven, Aldi, Batavia, Ill, USA). The indifferent barrel was back‐filled (Microfil 34 gauge tip, WPI) with 150 mM NaCl, 20 mM HEPES adjusted to pH 7.4 with 1 M NaOH. The ion‐sensitive barrel was back‐filled (Microfil 28 gauge tip, WPI) with 100 mM KCl, 20 mM HEPES adjusted to pH 7.2 with 1 M NaOH. The ion‐sensitive barrel was filled at the tip by front suction with a short (100–400 µm) column of K^+^ sensitive liquid ion sensor (K ionophore I Cocktail B, 99373, Sigma‐Aldrich). The ion‐sensitive microelectrode was connected to an HS‐2 x0.0001 MU headstage. The indifferent (reference) electrode was connected via an HS‐2A x0.1 LU headstage. Both headstages were connected to an Axoprobe 1B amplifier (Molecular Devices). The reference signal was subtracted from the ion‐sensitive signal and amplified 10×, then fed into a HumBug Noise Eliminator (Digitimer Ltd, Welwyn Garden City, UK) to remove mains frequency noise (50 Hz). The signal was acquired via a MiniDigi 1A using Axoscope 11 (Molecular Devices) at 1 kHz, which was subsequently reduced to 1 Hz for compatibility with CAP recordings. A motorised manipulator (EC1 60–0577 control unit with EC1 60–0571 standard motorised control manipulator: Harvard Apparatus, Holliston, MA, USA) was used for placement of the K^+^ sensitive microelectrode in the nerve. Electrodes were calibrated in a solution containing 120 mM NaCl and 20 mM HEPES, with KCl at concentrations of 3 mM, 9 mM or 30 mM. All electrodes were individually calibrated before and after the experiment, and only those showing stable, near Nernstian responses (above 50 mV) to decade changes in [K^+^] were used for experimental measurements. The average between the initial and final calibrations was used to evaluate experimental data. The [K^+^]_o_ was estimated as follows (Carlini & Ransom, 1990). For an order of magnitude increase in [K^+^] (3–30 mM), where *E*_1_ and *E*_2_ are the voltages recorded at the highest and lowest values of [K^+^], [K^+^]_1_ and [K^+^]_2_, respectively, the slope is defined as:

(1) $$\mathrm{Slope}\;=\frac{\left(E_{1}-E_{2}\right)}{\mathrm{lo}{g_{10}}^{\frac{{\left[K^{+}\right]}_{1}}{{\left[K^{+}\right]}_{2}}}}$$

In MONs superfused with 3 mM K^+^ aCSF, baseline [K^+^]_o_ was manually set to 3 mM as previously described (Forstl et al., 1982), and the voltage (*V*) recorded by the ion‐sensitive electrode was converted to [K^+^]_o_ according to:

(2) $${\left[K^{+}\right]}_{o}=3\;\mathrm{mM}\;\times 10^{\left(\frac{V}{\mathrm{slope}}\right)}$$

Measurement of interstitial space shrinkage was carried out using tetramethylammonium (TMA) as previously described (Brown et al., 2001).

The relationship between [K^+^]_o_ and the CAP illustrated in Figures 3c and 4b is described by the function:

(3) $$\mathrm{CAP}\;=\frac{\max}{1+\mathrm{ex}p^{\left[\frac{K_{0.5}-K}{\left(\mathrm{Slope}\right)}\right]}}\;$$

where max is the maximum CAP area, K_0.5_ is the [K^+^]_o_ at 0.5 × max, K is the [K^+^]_o_ or aCSF [K^+^] and Slope is a unitless parameter. Equation 3 can be rearranged to calculate the predicted [K^+^]_o_ associated with the CAP area (Figure 4c ) as follows:

(4) $${\left[K^{+}\right]}_{o}=-\left[\ln\left(\frac{\max}{\mathrm{CAP}}-1\right)\right]\times \;\mathrm{Slope}-K_{0.5}$$

### 2.5 Analysis of oscillations

Since the [K^+^]_o_ oscillations could be approximated as sin waves of the form:

(5) $${\left[K^{+}\right]}_{o}=\mathrm{amp}\times \sin\;\left(2\pi f\right)$$

Fast Fourier transform (FFT) was used to convert the time domain data to the frequency domain, from which individual spectral components, referred to as the power spectrum, could be identified, where amp is the amplitude and the period describes the duration of a complete cycle of the oscillation, where the frequency (*f*) is defined as 1/period (Figure 6a). In this study, we use minutes as the unit of time. The frequency of individual oscillations from multiple MONs was used to create a histogram (Figure 6b) to identify the frequency band (0.2–0.5 min^−1^) containing the oscillation data. The area under the curve (AUC) of this band was used to compare oscillation data (Figure 6c) with various treatments (e.g., varying aCSF; Figure 7) or pharmacological agents (Figures 8 and 9), and Student's paired *t*‐test was used to determine significance.

### 2.6 Data analysis and curve fitting

MON axon function was monitored quantitatively as the area under the CAP using Clampfit 11 (Molecular Devices), which represents the best measure of the number of active axons since currents generated by individual axons within a fibre tract are considered to sum linearly (Cummins et al., 1979). Post‐experimental calibrations, data manipulations, and fast Fourier transforms (FFT) were carried out using Microsoft Excel (Microsoft). Data were analysed with GraphPad Prism (GraphPad Software, Boston, MA, USA) using correlation, coefficient of variation, linear and non‐linear regression, histograms, area under the curve, *t*‐tests, and one‐way ANOVA to determine significance, where * indicates *P* < 0.05, ** *P* < 0.01, *** *P* < 0.001, **** *P* < 0.0001. Data are presented as means ± standard deviations (SD).

## 3 RESULTS

### 3.1 CAP oscillations

**FIGURE 1:**
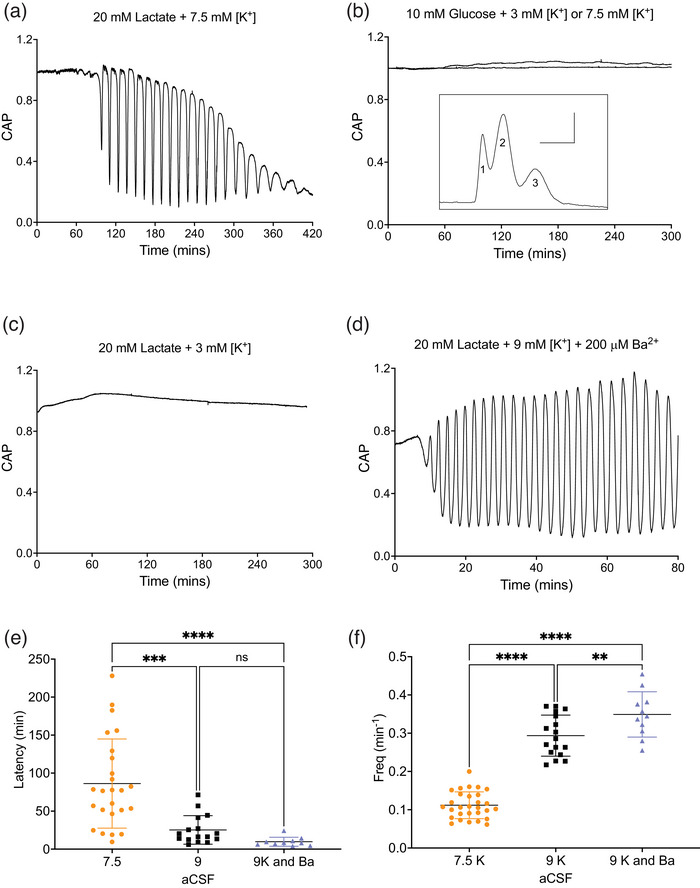
CAP area oscillations in stress aCSF. (a) Superfusing MONs with control aCSF maintained the CAP for over 6 h, whereas switching to 20 mM lactate and 7.5 mM K^+^ aCSF (starting at time 0 min) initiated delayed CAP oscillations in 50% of MONs tested (*n* = 29), with the amplitude of the initial oscillations becoming larger until the CAP was completely lost during each oscillation. There was a progressive decline in the baseline CAP area following the onset of oscillations. (b) The CAP was fully maintained for at least 5 h when superfused with 10 mM glucose and 3 mM K^+^ (control conditions: *n* = 10) or 10 mM glucose and 7.5 mM K^+^ (*n* = 4). and (c) 20 mM lactate and 3 mM K^+^ (*n* = 10). Inset in (b) shows an example CAP profile consisting of 3 clearly distinguishable peaks: scale bars are 1 ms and 1 mV. (d) In MONs superfused with 20 mM lactate, 9 mM K^+^ and 200 µM Ba^2+^, a decrease in the CAP area preceded oscillatory behaviour, which occurred in 64.7% of MONs tested (*n* = 11). (e) The latency to oscillation onset was significantly faster in MONs treated with 9 mM K^+^ (25.1 ± 18.2 min, *n* = 17) or 9 mM K^+^ + 200 µM Ba^2+^ aCSF (9.1 ± 5.6 min; *n* = 11) compared to 7.5 mM aCSF K^+^ (86.1 ± 57.3 min; *n* = 29). (f) The mean oscillation frequency was increased when MONs were incubated in 9 mM K^+^ (0.29 ± 0.05 min^−1^) or in 9 mM K^+^ + 200 µM Ba^2+^ (0.34 ± 0.06 min^−1^) compared to 7.5 mM aCSF K^+^ (0.11 ± 0.03 min^−1^). The oscillation frequency was internally consistent in each MON regardless of the composition of the stress aCSF. In 7.5 mM K^+^, the coefficient of variation for the oscillation frequency from 79 oscillations from 9 MONs was 7.45%, and in 9 mM K^+^ + 200 µM Ba^2+^, the coefficient of variation from 68 oscillations from 10 MONs was 4.57%.

In MONs superfused with control aCSF (10 mM glucose and 3 mM K^+^), the CAP is fully maintained for up to 6 h (Figure 1b). Switching to aCSF where the glucose was replaced by the carbon equivalent concentration of lactate (20 mM) and [K^+^] increased from 3 mM to 7.5 mM (stress aCSF) initially had no effect on the CAP, but after a delay, oscillations of the CAP area became evident when the CAP briefly decreased in amplitude before fully recovering. This cyclical process of CAP loss followed by recovery persisted for several hours (Figure 1a). Once the cycle of oscillation was established, the CAP failed completely during each oscillation, which coincided with a gradual decrease in the baseline CAP area. If the oscillations are ignored, the basic pattern is of temporary CAP maintenance, followed by a slow decrease in area over several hours (Figure 1a). When MONs were exposed to either control aCSF (Figure 1b), 10 mM glucose and 7.5 mM K^+^ aCSF (Figure 1b) or 20 mM lactate and 3 mM K^+^ aCSF (Figure 1c), the CAP was fully maintained for up to 5 h with no oscillatory behaviour, evidence that both the presence of an oxidative substrate *and* increased aCSF [K^+^] were required for oscillations. Since increased aCSF [K^+^] will directly activate the astrocytic NKA (Ransom et al., 2000; Rose & Ransom, 1996) and depolarise astrocytes, which will stimulate glycolysis (Ruminot et al., 2011; Sotelo‐Hitschfeld et al., 2015) and glycogenolysis (Choi et al., 2012; Hof et al., 1988; Xu et al., 2013), we reasoned that the delay in oscillation onset reflected the time taken for [K^+^]‐induced depletion of astrocytic energy reserves. We therefore accelerated oscillation onset by increasing aCSF [K^+^] to 9 mM (Figure 1e). To further accelerate the onset of the oscillations, we maintained aCSF [K^+^] at 9 mM and added 200 µM Ba^2+^ for the following reasons. The elevated aCSF [K^+^] will lead to K^+^ uptake into astrocytes, with estimates of [K^+^]_i_ increasing by up to 20 mM (Murakami & Kurachi, 2016). The change in electrochemical gradient will likely lead to K^+^ efflux via Kir channels as reported in both hippocampal and mouse optic nerve astrocytes after stimulus‐induced K^+^ influx (Bay & Butt, 2012; D'Ambrosio et al., 2002). Blocking such an efflux with Ba^2+^ (Walz et al., 1984) will lead to accumulation of intracellular +ve charge, depolarising the astrocyte membrane potential without the attenuating effects on CAP area that accompany [K^+^]_o_ elevation (Hopper et al., 2023). The effect of Ba^2+^ was to accelerate oscillation onset as predicted (Figure 1d). Thus, the initial serendipitous observation of oscillations of CAP area in MONs exposed to stress aCSF containing 7.5 mM K^+^ was optimised to accelerate oscillation onset (Figure 1e) and increase the probability of their occurrence. An additional benefit to this optimisation process was the increased frequency of CAP oscillations (Figure 1f), which offered a more robust signal during analysis.

### 3.2 CAP profile during oscillations

**FIGURE 2:**
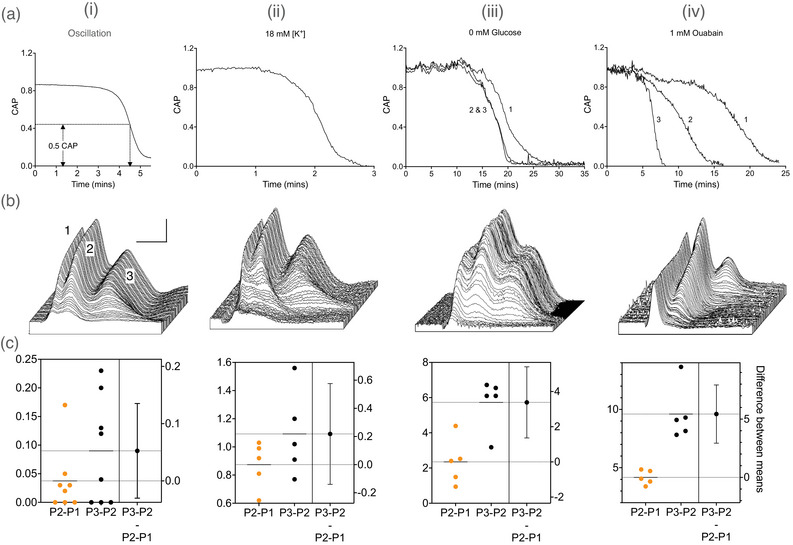
Comparison of the CAP profile during an oscillation and in response to metabolic insults. (ai) The CAP area during an oscillation showing a rapid fall. There was minimal difference in the latency to the individual peak fall, so the total CAP area is shown (*n* = 29). The time taken for the peaks to fall to 50% of the baseline value was measured as the latency. (bi) The loss of the CAP during an oscillation is represented by sequential CAPs, which are plotted on a waterfall graph, where the *z*‐axis denotes time, with the most recent CAPs towards the front of the stack. Scale bar: 1 mV and 1 ms. The numbers above the CAP represent the CAP peaks illustrated in Figure 1b (inset). (ci). The Estimation plot displaying the time difference of the latencies between P2 and P1 and between P3 and P2 at half of the amplitude of the CAP, where the units are minutes. The small values indicate a very close temporal relationship between the peaks. The difference between the two measures did not achieve significance (*t*‐test: *P* = 0.20). Data are illustrated for 18 mM K^+^ aCSF (*n* = 5) (ii), 0 glucose (*n* = 12) (iii), and ouabain (*n* = 10) (iv) in the same format as for the oscillations in Column (i). When MONs were exposed to aglycaemic aCSF in which glucose was removed, the CAP failed, but the loss of the 2nd and 3rd peaks preceded the loss of the 1st peak. In a similar manner, exposure to ouabain caused CAP failure, but the CAP peaks were lost in the following order: 3rd, then 2nd, then 1st. (c) The difference between the peaks was not significant for 18 mM K^+^ (*P* = 0.48), but did achieve significance for 0 glucose (*P* = 0.0048) and ouabain (*P* = 0.0001).

During an oscillation, the CAP took 90 s to fall (Figure 2ai). Judicious plotting of CAPs in a waterfall format shows how the profile changes during the oscillation. The CAP profile consists of three peaks, with distinct latencies, suggesting sub‐populations with varying diameters (Allen et al., 2006). All peaks fall at the same rate with a slight rightward shift in the latency (Figure 2bi). The profile of the CAP loss during an oscillation was reminiscent of CAP failure when aCSF [K^+^] was increased (Hopper et al., 2023), a comparison that invited closer inspection. A similar profile was seen (Figure 2bii) when MONs were exposed to 18 mM aCSF K^+^, suggesting the oscillatory effect on the CAP may result from changes in [K^+^]_o_. However, a rapid decrease in the CAP also occurs when exposing MONs to aglycaemic conditions when glucose is omitted from the aCSF (Brown et al., 2003). The pattern of CAP loss did not mimic that of the oscillation, with the second and third CAP peaks failing before the first peak (Figure 2biii). A similar pattern was seen in MONs exposed to ouabain, a blocker of the NKA, but again a staggered failure of the CAP peaks occurred (Figure 2biv). The similarity between the profile of CAP failure during the oscillation and exposure to increased aCSF K^+^ prompted us to record simultaneously the stimulus‐evoked CAP and interstitial [K^+^]_o_ from the same MON.

### 3.3 Interstitial [K^+^]_o_ recordings

**FIGURE 3:**
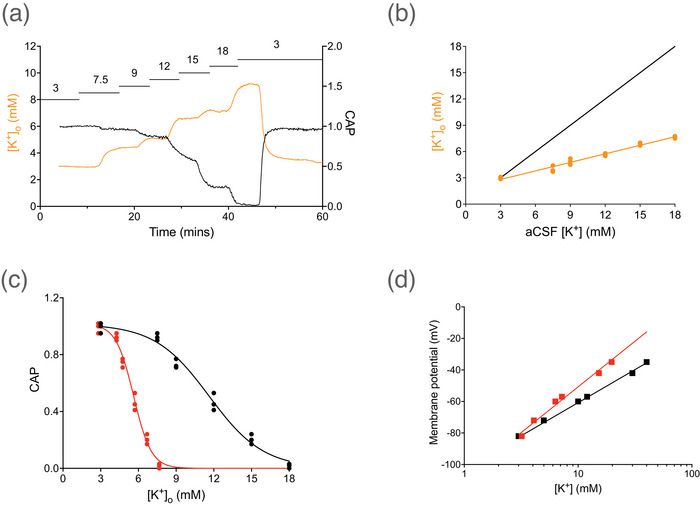
Relationship between aCSF [K^+^], [K^+^]_o_ and the CAP. (a) Simultaneous recording of CAP area (black line: right *y*‐axis) and [K^+^]_o_ (orange line: left *y*‐axis) recorded with a K^+^‐sensitive microelectrode represented by a single continuous experiment. The aCSF [K^+^] was progressively increased in a step‐wise manner, indicated in mM by the numbers above the horizontal bars, which depict the duration of exposure to the particular aCSF [K^+^]. (b) Plot of the measured [K^+^]_o_ versus the aCSF [K^+^], assuming a baseline of 3 mM when aCSF [K^+^] was 3 mM (orange circles, *n* = 3). The orange line is the best fit ([K^+^]_o_ = 0.32 × (aCSF [K^+^]) + 1.84) with an *R* ^2^ value of 0.98. The black line indicates the relationship where aCSF [K^+^] = [K^+^]_o_. (c) The aCSF [K^+^] versus CAP area (black circles) was described by Equation (3) (black line) where max = 1.016, K_50_ = 11.63 mM and slope = 2.163. Applying the correction factor described in (b) to the values of aCSF [K^+^] produced the relationship illustrated by the red circles and fit by Equation (3) (red line) where max = 1.016, K_50_ = 5.62 mM and slope = 0.703. (d). Historical data of astrocyte membrane potential recorded with a sharp microelectrode versus aCSF [K^+^] from *ex vivo* isolated rat optic nerve (Dennis & Gerschenfeld, 1969) recorded at 22–23°C. Data (black squares) have been measured from enlarged photocopies of the original paper and show a 42‐mV slope (black line) for a decade increase in log_10_(aCSF [K^+^]). Applying the correction factor described in (b) to the aCSF [K^+^] used in the paper allowed us to calculate a revised relationship, allowing for K^+^ buffering, between membrane potential and [K^+^]_o_, and depicted by the red squares. Curve fitting returned a Nernstian slope of 57.2 mV for these revised data, almost matching the theoretical value of 58 mV, with an *R* ^2^ value of 0.98.

A general assumption that is rarely tested in studies using *ex vivo* brain slices or isolated rodent optic nerve is that the interstitial [K^+^]_o_ recorded with K^+^‐sensitive microelectrodes accurately mirrors the aCSF [K^+^] (Leng & Shibuki, 1987). We investigated this by switching the aCSF [K^+^], whilst recording simultaneously the CAP and [K^+^]_o_ (Figure 3a). There is a wealth of information regarding [K^+^]_o_ in the MON with a consensus that 3 mM is an accurate reflection of [K^+^]_o_ when aCSF [K^+^] is 3 mM, but that even small increases in aCSF [K^+^] lead to activation of the astrocytic NKA (Ransom et al., 2000; Rose & Ransom, 1997), which acts to stabilise any variation in [K^+^]_o_ towards the baseline value of 3 mM. Consequently, we set our baseline value of [K^+^]_o_ to 3 mM in MONs bathed with 3 mM K^+^ aCSF (see Discussion). However, the measures of [K^+^]_o_ were significantly lower than aCSF [K^+^] when aCSF [K^+^] was increased above 3 mM, the relationship described by a linear function, which we refer to as the correction factor (Figure 3b). The reciprocal relationship between CAP area and [K^+^]_o_ is illustrated in Figure 3c, where the CAP decreased when aCSF [K^+^] exceeded about 8 mM, with the CAP completely lost in 18 mM K^+^: the CAP falls to 50% of its baseline area at about 11 mM aCSF K^+^. However, applying the correction factor described in Figure 3b (see Discussion) to the aCSF [K^+^] results in the relationship depicted by the red circles, where the response of the CAP to increasing [K^+^]_o_ is much steeper and occurs at lower values of [K^+^]_o_. Figure 3d is described in the figure legend.

### 3.4 CAP area and [K^+^]_o_ during oscillations

**FIGURE 4:**
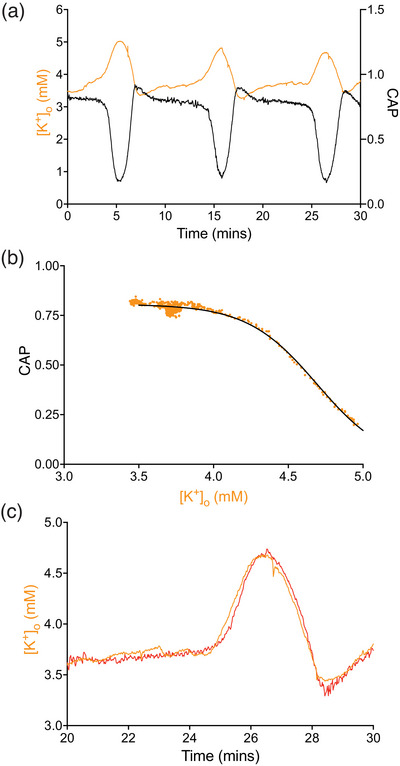
[K^+^]_o_ predicts CAP area during oscillations. (a) Simultaneous recordings of the CAP and [K^+^]_o_ from the same MON exposed to stress aCSF containing 20 mM lactate and 7.5 mM K^+^, spanning a period of 3 oscillations. The transient increases in [K^+^]_o_ coincide with the CAP fall, with the abrupt decrease towards baseline of the [K^+^]_o_ coinciding with the recovery of the CAP (*n* = 5). When correlations were carried out between the [K^+^]_o_ and the CAP from 15 separate experiments in MONs exposed to stress aCSF containing either 7.5 mM K^+^ or 9 mM K^+^, a mean value of −0.92 ± 0.07 (*n* = 15) was returned. (b) A plot of the CAP area versus [K^+^]_o_ from 5 to 10 min in the recording illustrated in (a) (orange circles), that is, during the rising phase of the CAP. The data are fit with Equation (3) (black line), where max = 0.93, K_0.5_ = 4.23 mM, and slope = 0.33. Equivalent fits carried out for oscillations from 15 separate MONs returned a mean *R* ^2^ value of 0.95 ± 0.09 (*n* = 15). (c) Superimposed traces of the measured [K^+^]_o_ (orange line) for the third oscillation in (a), with the computed [K^+^]_o_ according to Equation (4) (red line), illustrate an almost exact match with a correlation of 0.98. Equivalent traces from 15 separate MONs returned a correlation of 0.93 ± 0.12.

Simultaneous recordings of the CAP and interstitial [K^+^]_o_ give a clear indication as to the potential mechanism that initiates the CAP oscillations. During a cycle of three complete oscillations, when the stress aCSF comprised 20 mM lactate and 7.5 mM K^+^, there was a close temporal relationship between elevations in [K^+^]_o_ and decreases in the CAP, where the lowest point of the CAP oscillation coincided with the highest value of [K^+^]_o_. Reduction of [K^+^]_o_ towards baseline coincides with CAP recovery (Figure 4a). The correlation of close to −1 between the two variables indicates an inverse relationship, where an increase in one variable coincides with a decrease in the other, although no direct causation can be implied. Based on this converse correlation, we estimated the changes in [K^+^]_o_ that would produce the variations in CAP area that occur during an oscillation. To achieve this required plotting [K^+^]_o_ against the full range of the CAP area: this is illustrated in Figure 4b for a time span of 5–10 min in Figure 4a, which encompasses the rising phase of a CAP oscillation. Fitting the data with an appropriate function (Equation (3)) produced the parameters required to calculate CAP area associated with [K^+^]_o_: note the similarity between this ‘instantaneous’ relationship between CAP and [K^+^]_o_ and the ‘steady state’ relationship illustrated in Figure 3c. Rearranging Equation (3) allows estimation of the [K^+^]_o_ predicted from the CAP (Equation (4)), and when superimposed on the measured [K^+^]_o_ shows an identical profile, a strong indication that changes in [K^+^]_o_ govern the CAP area during the oscillatory behaviour (Figure 4c).

### 3.5 Rate of K^+^ fluxes

**FIGURE 5:**
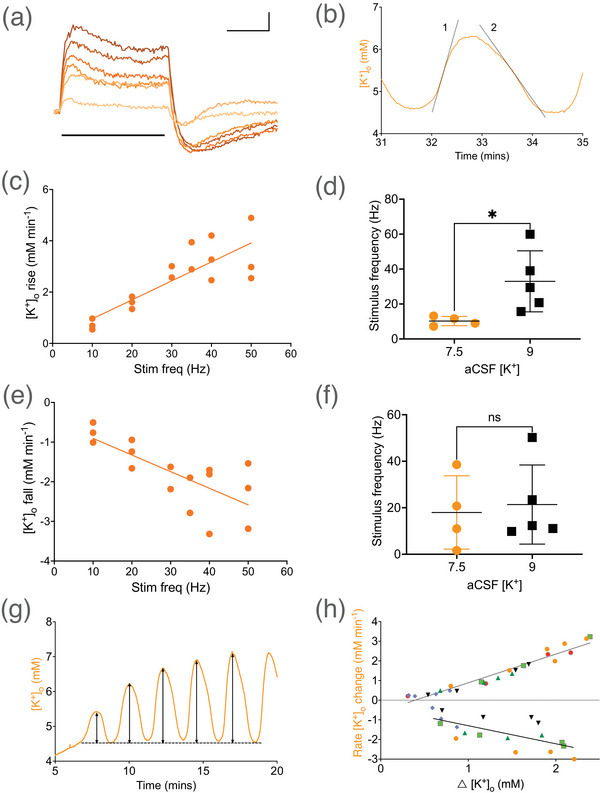
[K^+^]_o_ response to 2‐min periods of high frequency stimulus. (a) The recordings from an individual MON are overlaid to align temporally with the period of stimulus indicated by the horizontal black bar. The stimulus frequencies ranged from 10 to 50 Hz, the largest [K^+^]_o_ corresponding to the highest stimulus frequency. Scale bars are 0.5 mM [K^+^]_o_ and 1 min. (b) Recording of a [K^+^]_o_ oscillation, where the lines indicate where the rates of increase and decrease were measured. (c) The rate of [K^+^]_o_ increase at the onset of the stimulus versus stimulus frequency (*n* = 3). The relationship was described by y = 0.075x + 0.25 (*R* ^2^ = 0.69). (d) The rates of [K^+^]_o_ increase during an oscillation in 7.5 mM (1.02 ± 0.20 mM min^−1^, *n* = 4) or 9 mM (2.72 ± 1.31 mM min^−1^, *n* = 5) aCSF K^+^ and Ba^2+^ corresponded to stimulus frequencies of 10.3 Hz (*n* = 4) or 33.1 Hz (*n* = 5, *P* = 0.038), respectively. (e) The rate of [K^+^]_o_ decrease at the offset of the stimulus versus stimulus frequency (*n* = 3) was described by y = –0.042x – 0.50; *R* ^2^ = 0.534. (f) The rates of [K^+^]_o_ decrease during an oscillation in 7.5 mM (–1.22 ± 0.71 mM min^−1^, *n* = 4) or 9 mM (–1.40 ± 0.71 mM min^−1^, *n* = 5) aCSF K^+^ and 200 µM Ba^2+^ corresponded to stimulus frequencies of 16.9 Hz (*n* = 4) or 21.1 Hz (*n* = 5, *P* = 0.76), respectively. (g) During the development of the oscillations, the rate of [K^+^]_o_ increase or decrease was measured relative to the [K^+^] gradient measured as the peak [K^+^]_o_ minus the baseline value. (h) Sequential measurements of developing oscillations from 6 MONs (with each MON depicted as a distinct colour) show a linear relationship between Δ[K^+^]_o_ and the rate of increase (*R* ^2^ = 0.94) or decrease (*R* ^2^ = 0.46) in [K^+^]_o_.

MONs exposed to stimulus of increasing intensities for periods of 2 min showed the same basic pattern, of an initial rapid increase in [K^+^]_o_ followed by a plateau or even a fall in [K^+^]_o_ followed by a rapid post‐stimulus undershoot (Figure 5a). Plotting the rate of rise (Figure 5c) or rate of decrease (Figure 5e) of [K^+^]_o_ post‐stimulus versus stimulus frequency produced linear relationships. From these relationships, and from measuring the rates of [K^+^]_o_ increase or decrease during oscillations in stress aCSF (Figure 5b), we were able to estimate the stimulus frequency required to produce comparable changes in [K^+^]_o_ (Figure 5d, f), with the aim of highlighting the rapid K^+^ fluxes that occur during the oscillations. Since the data in Figure 5d, f suggest the [K^+^] gradient between the aCSF and [K^+^]_o_ has a more profound bearing on the rising phase of the oscillation than the falling phase, we used the nascent oscillations that occur as a prequel to fully blown oscillations to investigate how the [K^+^] gradient relates to the change in [K^+^]_o_ during the rising and falling phase of an oscillation. The rate of [K^+^]_o_ change at the steepest part of the slope (Figure 5b) was plotted against the difference between the peak and baseline [K^+^]_o_ (Figure 5g). Although both the increase and decrease of [K^+^]_o_ were linearly related to the [K^+^] gradient, the increase is more accurately predicted by the gradient than the decrease (Figure 5h), reflected in a larger *R* ^2^ value of the fit.

### 3.6 Analysis of oscillatory behaviour

**FIGURE 6:**
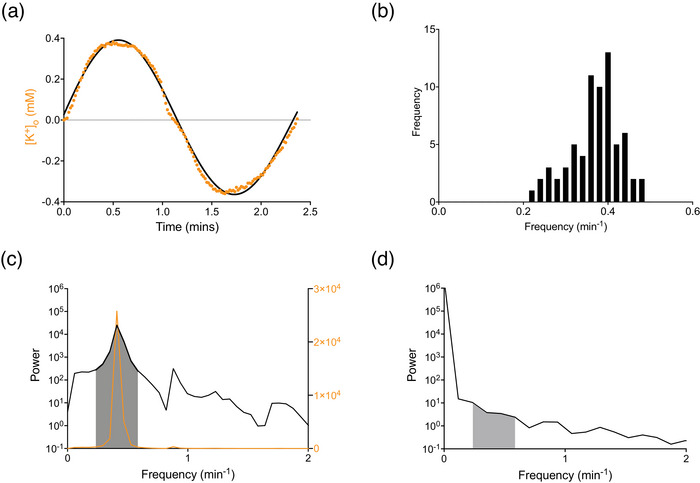
Analysis of oscillations. (a) The oscillations of [K^+^]_o_ initiated by stress aCSF containing 9 mM K^+^, 20 mM lactate and 200 µM Ba^2+^ could be fit by a sine wave (Equation (5)). In the example shown, a single cycle is illustrated with a frequency of 0.42 min^−1^, an amplitude of 0.40 mM, and the *R* ^2^‐value of the fit is 0.992. (b) The result of imposing the fast Fourier transform. This resolves the signal into spectral components, allowing identification of the principal frequency of the oscillations, between 0.2 and 0.5 min^−1^. Data of 69 individual oscillations from 10 MONs. (c) Power spectra density of the data illustrated in Figure 1d, showing the dominant peak between 0.2 and 0.5 min^−1^. The shaded area depicts the AUC used for comparative t‐tests. The orange line is the data represented on a linear scale (right *y*‐axis), clearly identifying the peak frequency. (d) Power spectra density showing data that do not contain oscillations, resulting in a peak frequency of 0 min^−1^.

**FIGURE 7:**
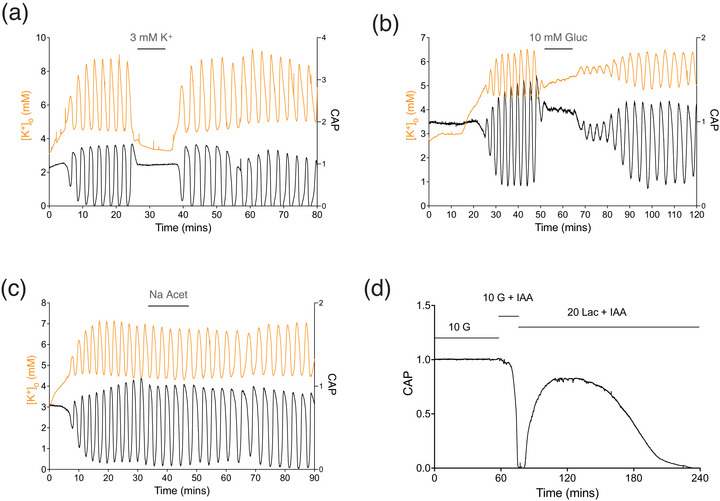
Interrupting the oscillations. (a) Substituting 7.5 mM K^+^ with 3 mM in MONs superfused with 20 mM lactate halted the oscillations (*P* = 0.0017), an effect which was reversible upon reintroduction of 7.5 mM K^+^ (*n* = 5). (b) A similar reversible effect occurred when 20 mM lactate was replaced with 10 mM glucose (*P* = 0.026) in the presence of 7.5 mM K^+^ (*n* = 6). (c) The oscillations were not halted when the oxidative substrate Na^+^ acetate was added to the aCSF (*n* = 4, *P* = 0.28). (d) MONs were bathed in 10 mM glucose aCSF to establish the baseline CAP area. Exposure to 1 mM IAA caused a rapid CAP failure that was temporarily rescued when 20 mM lactate was superfused, but the recovery was temporary, and the CAP slowly failed over a period of 2 h (*n* = 22), falling to half the recovery area in 91.2 ± 20.4 min. Note the similarity of the shape of the CAP baseline to the CAP baseline in Figure 1a.

The [K^+^]_o_ oscillations could be approximated as sine waves (Figure 6a), making them suitable for Fourier transform analysis, which consists of resolving the component frequencies present in the oscillation data. The analysis of all subsequent data in this paper used stress aCSF consisting of 20 mM lactate, 20 mM K^+^ and 200 µM Ba^2+^. Measurement of individual oscillations, which were plotted as a histogram, showed the frequency of the oscillations lay between 0.2 and 0.5 min^−1^ (Figure 6b). The result of the fast Fourier transforms of data displayed in the time domain (e.g., Figure 6d) is expressed as a power spectral density (Figure 6c), which isolates the dominant frequency. The shaded area represents the area under the curve for the frequency band identified in Figure 6b. The data are shown on a log_10_ scale to be consistent with the baseline data (Figure 6c), but are also shown on a linear scale (orange trace: right *y*‐axis) to clearly indicate the dominant frequency. Fourier transform of baseline data (e.g., Figure 1c), where there is no obvious oscillatory behaviour, returns 0 as the dominant frequency (Figure 6d). Comparison between the AUC for data, as illustrated in Figure 7a–c, was used to determine any differences between oscillatory data versus treatment.

### 3.7 Interrupting the oscillations

Once oscillatory behaviour had been initiated by exposure to stress aCSF, the oscillations could be halted by either reducing the aCSF [K^+^] to 3 mM (Figure 7a) or by replacing the 20 mM lactate with 10 mM glucose (Figure 7b). The oscillations could also be halted (data not shown) by adding the glycolytic substrates mannose (10 mM: *n* = 4; *P* = 0.022) or fructose (20 mM: *n* = 4; *P* = 0.002). Resumption of oscillations occurred upon reintroduction of the stress aCSF. However, the oxidative substrate sodium acetate did not halt the oscillations (10 mM, Figure 7c), suggesting inadequate transport across the astrocyte membrane. This was supported by the inability of sodium acetate to support CAP conduction when it replaced glucose in control aCSF (*n* = 3, data not shown). The slow decrease in the CAP baseline illustrated in Figure 1 suggests that lactate cannot support astrocytic function, particularly its ability to buffer [K^+^]_o_, for extended periods. This was further investigated by experiments where we superfused MONs with glucose to establish a baseline CAP area before adding 1 mM iodoacetate (IAA), an inhibitor of the glycolytic enzyme glyceraldehyde 3‐phosphate dehydrogenase (Leppanen & Stys, 1997), which will prevent the tissue from benefitting from glucose or glycogen, and the CAP fell abruptly after about 15 min. The CAP could be recovered by including 20 mM lactate with the IAA, but the CAP recovery was not maintained and began to fall after about an hour, with a slow failure occurring over the next 2 h (Figure 7d).

### 3.8 The effect of TTX on the oscillations

**FIGURE 8:**
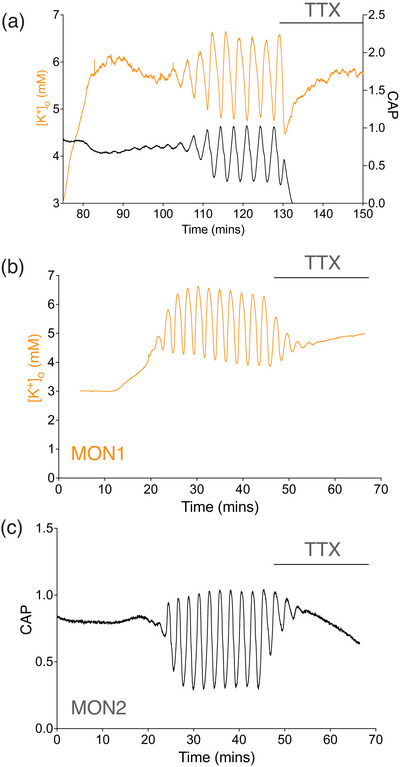
TTX blocks the CAP and [K^+^]_o_ oscillations recorded from the same MON. (a) The stimulus‐evoked CAP and [K^+^]_o_ recorded from the same MON showed reciprocal oscillatory behaviour, where the oscillations started a short time after introducing the stress aCSF, preceded by an increase in [K^+^]_o_ from a baseline value of 3 mM. Addition of 100 nM TTX to the aCSF caused an abrupt halt in both the CAP and [K^+^]_o_ oscillations, with the CAP area rapidly falling to zero (*P* = 0.019: *n* = 8). (b, c) TTX blocks the CAP and [K^+^]_o_ oscillations recorded from separate MONs. A pair of MONs from the same mouse were placed in the recording chamber, and a K^+^ sensitive microelectrode was inserted into MON1, which was not stimulated, whereas MON2 was inserted into suction electrodes, and the stimulus evoked CAPs at 1 Hz. Switching to stress aCSF (20 mM lactate, 9 mM K^+^ and 200 µM Ba^2+^) initiated oscillations in both nerves, [K^+^]_o_ oscillations in MON1 and CAP oscillations in MON2. The periodicity of the oscillations was identical (0.363 ± 0.088 vs. 0.355 ± 0.090 min^−1^; *P* = 0.66), and both were halted when 10 nM TTX was added to the aCSF, the CAP area slowly falling and the [K^+^]_o_ steady (*n* = 5).

We investigated the relationship between the stimulus‐evoked CAPs and the [K^+^]_o_ oscillations by simultaneously recording the CAP from MONs in which we inserted a K^+^ electrode. Upon exposure to stress aCSF containing 20 mM lactate, 9 mM K^+^ and 200 mM Ba^2+^ there was a simultaneous increase in [K^+^]_o_ and a fall in CAP amplitude, followed by the onset of reciprocal synchronised oscillations (Figure 8a). When 100 nM TTX was added, the CAP and the [K^+^]_o_ oscillations were abruptly halted, an intriguing result that seemingly linked stimulus‐evoked CAPs to the [K^+^]_o_ oscillations and suggested that the elevations in [K^+^]_o_ that occur during the oscillations may be a result of K^+^ efflux from axons firing CAPs. However, this assumption warranted closer inspection since the 1 Hz baseline CAP stimulus rate is far slower than the frequencies reported in Figure 5. We deemed it necessary to separate the stimulus‐evoked CAP firing from the [K^+^]_o_. To accomplish this, we placed a pair of optic nerves from the same mouse in the recording chamber. We inserted a K^+^ microelectrode into the first nerve (MON1, Figure 8b), which was not stimulated, and attached stimulating electrodes to the second nerve (MON2, Figure 8c). Upon switching to the stress, aCSF oscillations were initiated in both nerves at comparable times, which cycled with equivalent periodicity until the addition of 10 nM TTX, a half‐maximal blocking dose of TTX in MON CAP (Chen et al., 2002). TTX caused the magnitude of the oscillations to decrease until, after about 5 min, the [K^+^]_o_ and CAP oscillations were abolished, and the CAP amplitude began to fall. The results demonstrate that the stimulus‐evoked CAP was not required to initiate the oscillations, the oscillation periodicity is independent of stimulus frequency, but that axonal TTX‐sensitive action potentials were required to sustain the CAP.

### 3.9 Inhibiting the axonal NKA blocks the oscillations

**FIGURE 9:**
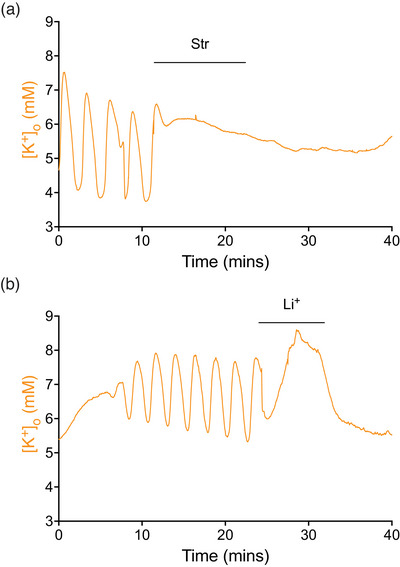
Inhibiting the axonal NKA blocks the oscillations. (a) Addition of 50 µM strophanthidin halts the [K^+^]_o_ oscillations (*n* = 3; *P* = 0.0132). (b) Replacing NaCl with LiCl blocks the [K^+^]_o_ oscillations (*n* = 3; *P* = 0.0348).

Application of 50 µM strophanthidin, a blocker of the axonal, but not astrocytic NKA (Ransom et al., 2000), inhibited the oscillations in MONs exposed to stress aCSF (Figure 9a), clearly indicating a key role for the axonal NKA in maintenance of the oscillations. Lithium chloride can be used to substitute for NaCl since Li^+^ is permeable at the voltage‐gated Na^+^ channel; thus, it will not block the CAP, but intra‐axonal Li^+^ inhibits the axonal NKA (Ploeger, 1974). Replacing NaCl with LiCl blocked the [K^+^]_o_ oscillations (Figure 9b).

## 4 DISCUSSION

The compound action potential oscillations we describe were a serendipitous discovery when studying the ability of astrocytes to function when exposed to the metabolic stress aCSF. We investigated their underlying mechanism, which revealed novel aspects of interstitial K^+^ buffering in the mouse optic nerve. The unique oscillatory behaviour is activated when astrocytes are bathed in supra‐physiological aCSF [K^+^] and starved of energy substrate, rendering them incapable of adequately buffering [K^+^]_o_ and precipitating a rapid increase in [K^+^]_o_ towards the aCSF [K^+^], which was accompanied by a loss of the CAP. The rise in [K^+^]_o_ was reversed by intermittent activation of the axonal NKA, causing a rapid fall in [K^+^]_o_. Subsequently, in the absence of either axonal or astrocytic NKA activity, the [K^+^]_o_ rose rapidly and approached aCSF [K^+^], and the oscillatory cycle continued. The results reveal a clear separation of the astrocytic and axonal [K^+^]_o_ buffering in MON, where astrocytes dominate at rest, but axons dominate during the oscillations, clearly demonstrating the powerful buffering capacity present in the MON when faced with supra‐physiological [K^+^]_o_.

### 4.1 Interstitial [K^+^]_o_ buffering

Perhaps the most surprising aspect of this study is the relatively modest increase in [K^+^]_o_ that initiates the oscillations (Figure 4a), compared to the large stimulus‐induced [K^+^]_o_ elevations that can approach 12 mM in white and grey matter (Ballanyi et al., 1987; Connors et al., 1982; Ransom et al., 2000). A summary of rodent in vivo [K^+^]_o_ in a variety of brain regions is available (Rasmussen et al., 2020), typically between 3 and 5 mM, values adopted for aCSF [K^+^] in *ex vivo* experiments. Ex vivo measures of [K^+^]_o_ within grey matter brain slices or rodent optic nerve preparations using ion sensitive microelectrodes (ISM) are complicated by the calibration process, since moving the ISM through the air between the calibration solutions and the superfusion chamber can introduce DC shifts in the indifferent barrel that interfere with the accuracy of the recordings (Forstl et al., 1982). Under these conditions, the voltage on the amplifier is manually adjusted to reflect aCSF [K^+^] (Brown et al., 2001; Connors & Ransom, 1984; Connors et al., 1982; Forstl et al., 1982; Hoppe et al., 1991; Ransom et al., 2000), or changes in [K^+^]_o_ are reported relative to a baseline of 0 mM, rather than absolute concentrations (Bay & Butt, 2012).

In the mid‐1960s, two papers were published that have profound implications for the current study. The first proposed a role for astrocytes in buffering [K^+^]_o_ in mammalian brain (Hertz, 1965), and the second demonstrated that amphibian glial cell membrane potentials respond to variations in bathing solution K^+^ according to Nernstian predictions (Kuffler et al., 1966). An assumption that is rarely tested is that aCSF [K^+^] increases are accurately reflected in ISM measures of [K^+^]_o_. In the present study on MON, and in an equivalent study in isolated rat neurohypophysis (Leng & Shibuki, 1987), a disparity emerges between [K^+^]_o_ and aCSF [K^+^] when aCSF [K^+^] is increased beyond 3 mM. We plotted the [K^+^]_o_ versus aCSF [K^+^] and fit the relationship with a linear correction factor, which we applied to aCSF [K^+^] to provide an accurate estimate of the true [K^+^]_o_ value. Thus, researchers should be aware that aCSF [K^+^] beyond baseline values of 3 mM is not reflected in interstitial [K^+^]_o_ increases in *ex vivo* tissue, due to powerful K^+^ buffering. This disparity offers a convincing explanation for a historical anomaly, where attempts to reproduce the results of the amphibian study in mammalian glial cells (astrocytes) in rodent optic nerve or cat cortex routinely returned a slope of about 40 mV between the membrane potential and log_10_(aCSF K^+^) (Dennis & Gerschenfeld, 1969; Pape & Katzman, 1972; Ransom & Goldring, 1973), implying a marginal permeability to ions other than K^+^ (Powell & Brown, 2021). However, the exclusive permeability of rodent brain astrocytes to K^+^ was confirmed in a study that recorded both intra‐ and extracellular potentials in addition to [K^+^]_o_ in a single location in the cat spinal cord, where modest [K^+^]_o_ increases, evoked by electrical stimulus, elicited Nernstian responses in astrocyte membrane potential (Lothman & Somjen, 1975). The reconciling of historic data with the effects of powerful buffering on [K^+^]_o_, described in the legend to Figure 3d where the correction factor was applied to aCSF [K^+^], offers a convincing explanation for a decades‐old anomaly where astrocyte membrane potential did not respond in a Nernstian manner to changes in aCSF [K^+^].

The evolution of the mammalian central nervous system, restricted by the confines of a rigid skull, prioritised bit rate transfer (Perge et al., 2009), leading to small axons (∼1 µm in diameter) separated by a very narrow interstitial space. The concentrating effect of a small interstitial space upon K^+^ efflux from active neurones would depolarise all neural membranes (Hille, 2001), compromising electrical activity, as demonstrated in our MON model where the CAP starts to fall when [K^+^]_o_ exceeds 3.9 mM and is completely lost at about 5.5 mM (Figure 4b), indicating a steep voltage dependence of axonal Na^+^ channel inactivation. To combat such events powerful buffering evolved to limit [K^+^]_o_ increases, the numerous transmembrane K^+^ transport processes (Larsen et al., 2014) coupled with syncytial astrocytes interconnected via gap junctions (Nedergaard et al., 2003), ensuring differences between astrocyte membrane potential and K^+^ equilibrium potential led to favourable dispersion of K^+^ from regions of high to low concentration (Kofuji & Newman, 2004). Our data suggest very powerful buffering that can limit increases in [K^+^]_o_ when aCSF [K^+^] is increased, at least in the short term in *ex vivo* models such as rodent optic nerve, which is evident as the prequel to the oscillations when [K^+^]_o_ increases on introducing stress aCSF (Figure 4a). This buffering capacity ensured aCSF [K^+^] did not equilibrate with the [K^+^]_o_, the astrocytes, which occupy about 30% of the optic nerve volume (Perge et al., 2009), acting as a sink to sequester K^+^. The modest increases in [K^+^]_o_ we report are reflected in a variety of studies where physiological stimulus increases [K^+^]_o_ by up to 0.5 mM (Somjen, 1979), and in rodent sleep studies where [K^+^]_o_ drives sleep state changes from awake (3.8 mM) to quiet awake (4.3 mM) to active awake (4.8 mM) (Rasmussen et al., 2020).

### 4.2 Metabolic stress

The CAP oscillations were initially observed when investigating the effects on astrocyte function of increasing aCSF [K^+^] and replacing a glycolytic fuel (glucose) with an oxidative substrate (lactate). Introducing aCSF containing [K^+^] in excess of 7.5 mM will increase the activity of the membrane‐bound astrocyte NKA, an energy‐demanding process (Ransom et al., 2000; Rose & Ransom, 1996); it will also stimulate astrocytic glycolysis (Ruminot et al., 2011; Sotelo‐Hitschfeld et al., 2015) and glycogenolysis (Choi et al., 2012; Hof et al., 1988; Xu et al., 2013). Inclusion of Ba^2+^ will depolarise astrocyte membrane potentials (Walz et al., 1984), which will further deplete astrocytic energy reserves. The rapid onset of oscillations under this condition (9 min) versus the 20 min latency to CAP failure on removal of glucose from the aCSF (Brown et al., 2003) demonstrates just how rapidly the stress aCSF depletes astrocyte energy reserves. The ability of lactate to support astrocyte energy demands is an unexplored area, since the principal focus for the last three decades has been on lactate export from astrocytes supporting neural function (Chih & Roberts, 2003; Machler et al., 2016; Magistretti & Allaman, 2018; Pellerin & Magistretti, 2012), although lactate uptake into astrocytes is reported at supra‐physiological concentrations up to 40 mM (Gandhi et al., 2009). Astrocytes contain 70% of MON mitochondria (Perge et al., 2009), but even in the presence of oxygen, glycolysis is the preferred catabolic pathway (Allen et al., 2005; Takahashi, 2021). The metabolic cooperativity, where astrocytes forsake interstitial glucose, metabolising instead their reserves of glycogen, thus sparing glucose for neural use (DiNuzzo et al., 2010), likely applies to the conditions described in this current paper, where axons exclusively use exogenously supplied lactate and astrocytes metabolise glycogen. This preference/reliance on glycolysis may be explained by the very fine processes that extend from the astrocyte soma (Oberheim et al., 2009), and which are too narrow to accommodate mitochondria, but which have a high energy requirement given their active contribution to synaptic activity (Haydon & Carmignoto, 2006). This failure of the astrocytic NKA, even when lactate is present in the bathing aCSF, is convincing evidence that astrocytes cannot efficiently metabolise lactate present in the interstitial space and supports the decades‐long assertion that astrocytes are predominantly glycolytic even in the presence of abundant oxygen (Pellerin & Magistretti, 2003), consistent with our experiments in which the glycolytic substrates glucose, mannose and fructose halted the oscillations, whereas the oxidative substrate lactate did not. The oxidative substrate sodium acetate (Dienel & Cruz, 2006) did not halt oscillations, suggesting it cannot be efficiently taken up and metabolised by astrocytes. This is supported by the cellular expression of monocarboxylate transporters, proteins that facilitate lactate flux across cell membranes. Astrocytes express MCT4, consistent with lactate efflux, whereas axons express MCT2, consistent with lactate influx (Machler et al., 2016). In physiological [K^+^]_o_ lactate cannot support axon conduction for extended periods (Figure 7d), a reasonable assumption being that astrocyte buffering fails under such circumstances, with the resulting increase in [K^+^]_o_ inhibiting CAP conduction Supporting Information.

### 4.3 Oscillations

**FIGURE 10:**
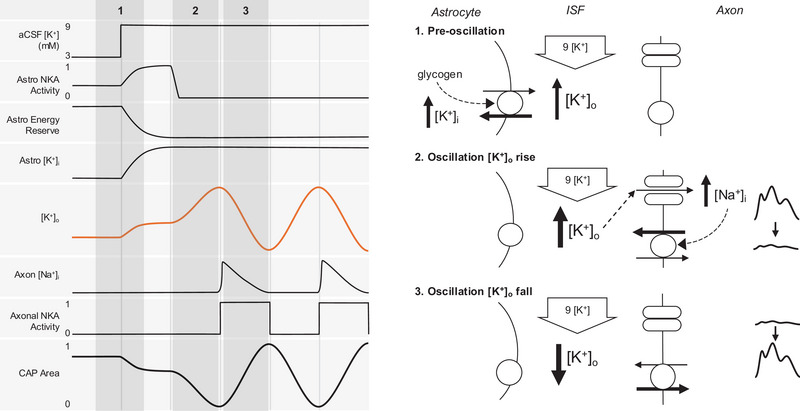
Model of [K^+^]_o_ oscillations in MON. A graphical illustration of the sequence of events that precipitate the oscillations. (a) Under pre‐oscillation equilibrium, on switching to 9 mM K^+^ and 20 mM lactate aCSF, the astrocytic NKA is activated (causing increased [K^+^]i), which requires additional energy provided by the limited glycogen stores in the astrocytes. (b) Depletion of these reserves renders the astrocyte unable to buffer [K^+^]_o_ since they cannot utilise exogenously applied lactate, and [K^+^]_o_ approaches aCSF [K^+^] causing CAP failure. (c) At the [K^+^]_o_ peak, axonal voltage‐gated Na^+^ channels open, resulting in increased Na^+^ influx, which activates the axonal NKA, facilitating [K^+^]_o_ decrease and recovery of the CAP. The continued presence of high [K^+^]_o_ causes cycling of the oscillatory process.

The transient cyclical fluctuations in [K^+^]_o_ that we observed meet the three criteria that define an oscillation; first, there is an excitatory drive that activates the system; second, there is an inhibitory feedback drive that returns the system towards steady state; and third, there is a delay in the inhibitory feedback loop (Friesen & Block, 1984). Analysis of oscillations can offer insights into mechanisms underlying the coordination of synchronous events (Donoghue et al., 2022). The oscillations commence with a rapid increase in [K^+^]_o_, which provides indirect estimates of the astrocyte contribution to [K^+^]_o_ buffering, since the rate of [K^+^]_o_ increase (2.72 mM min^−1^) during the upstroke of the oscillation must equal the rate of K^+^ influx into astrocytes immediately prior to the oscillation. That the [K^+^]_o_ oscillations start in the absence of stimulus‐evoked CAPs is evidence of an astrocyte origin. The dependence of the rate of [K^+^]_o_ increase during the upstroke of the oscillation on the [K^+^] gradient is consistent with a flux of K^+^ from the aCSF to the interstitial space, as is the approach of [K^+^]_o_ towards aCSF [K^+^], but never exceeding it, which could be the case if the upstroke were the result of rapid cellular K^+^ efflux. The experimental evidence suggests the inhibitory drive of the oscillations arises from activation of axonal NKA. The oscillations are inhibited by TTX in MONs that were not stimulated (Figure 8b), consistent with axonal action potential involvement, supported by the inhibition of the oscillations by the selective axonal NKA blocker strophanthidin (Ransom et al., 2000). Replacing NaCl with LiCl provides further evidence of a requirement for axonal action potentials in sustaining the oscillations, as Li^+^ permeates the voltage‐gated Na^+^ channel but inhibits the NKA (Ploeger, 1974). An increase in [K^+^]_o_ from 3 to 7 mM, assuming a constant [K^+^]_i_ of 120 mM, depolarises the K^+^ reversal potential by about 20 mV, where the axonal membrane potential can be expected to depolarise more than 10 mV (Powell & Brown, 2021). Thus, a scenario emerges of axonal accumulation of Na^+^, facilitated by Na^+^ influx via TTX‐sensitive voltage‐gated Na^+^ channels activated when [K^+^]_o_ rises to a critical concentration sufficient to depolarise the axon membrane potential to Na^+^ channel threshold (Zang & Marder, 2021). Additional evidence comes from studies of high frequency stimulus on *ex vivo* brain slice and optic nerve preparations bathed in 3 mM K^+^ aCSF, where stimulus leads to [Na^+^]_i_ accumulation in axons (Zang & Marder, 2021) that activates the NKA, the post‐stimulus dips in [K^+^]_o_ below baseline (Figure 5a) evidence of the axonal NKA activity (Bay & Butt, 2012; D'Ambrosio et al., 2002; Hopper et al., 2023; Ransom et al., 2000), since astrocytic NKA would simply return the [K^+^]_o_ to the baseline value of 3 mM. During experiments with 7.5 mM aCSF K^+^, small abortive oscillations emerged that did not evolve into full‐blown oscillations, most likely due to the axonal membrane depolarisation just failing to reach Na^+^ channel threshold. During the falling phase of the oscillations, only the axonal NKA is active at buffering [K^+^]_o_. It must be appreciated that there is a constant flux of K^+^ from the aCSF to the interstitial space of 2.72 mM min^−1^, which must be exceeded for any [K^+^]_o_ decreases to occur. Thus, the fall in [K^+^]_o_ of 1.40 mM min^−1^ is evidence of extremely powerful and previously unreported axonal K^+^ buffering. The [K^+^]_o_ nadir signifies that axonal NKA activity has returned axonal [Na^+^]_i_ to baseline levels, removing the stimulus for NKA activation, leaving the MON with no buffering capacity. Under these conditions, the [K^+^]_o_ rises rapidly towards aCSF [K^+^] until the axonal NKA is activated as described above, and the oscillatory cycle continues (Figure 10). Thus, during sustained oscillations, intermittent axonal NKA activation is the sole means of activity buffering [K^+^]_o_ in the MON, a unique state since astrocytes have been so clearly associated with [K^+^]_o_ buffering for six decades.

Other possible mechanisms explaining the oscillations were explored using a variety of pharmacological agents. The metabolic aspect coupled to K^+^ flux would suggest a role for K_ATP_ channels, where depletion of astrocytic ATP opens K_ATP_ channels, leading to K^+^ efflux, consistent with the increase in [K^+^]_o_ that defines the start of the oscillations. However, tolbutamide (*n* = 3; *P* = 0.19) had no effect on the oscillations (Weston et al., 1990). Furosemide (*n* = 9; *P* = 0.17) had no effect on the oscillations, suggesting the NKCC1 transporter had negligible involvement (Hamann et al., 2010; Zeuthen & Macaulay, 2012). The [K^+^]_o_ changes could have been due to cyclical shrinkage and expansion of the extracellular space that would accompany significant trans‐membrane K^+^ fluxes, but using the standard TMA technique (Sykova & Nicholson, 2008), only marginal changes of 4.7 ± 1.4% in [TMA^+^]_o_ during 24 oscillations from three MONs were recorded (*n* = 3), which would not explain the oscillations.

### 4.4 Conclusion

The serendipitous nature of the discovery of the oscillations described in this paper should not detract from the revelations relating to energy substrate use and buffering we describe. Whereas astrocytes are incapable of efficiently metabolizing exogenously supplied lactate, axonal activity flourishes, the most convincing demonstration yet of glycolytic astrocytes and oxidative axons in intact central white matter. The power of astrocyte and axonal buffering are equally impressive, their combined contribution ensuring limited [K^+^]_o_ increases and rapid recovery when [K^+^]_o_ equilibrium is disturbed, and entirely consistent with these prescient observations from pioneering studies of mammalian central nervous system K^+^ buffering; ‘Indeed, reasonable estimates suggest interstitial [K^+^]_o_ increases of up to 4 mM, but never beyond 5 mM, during physiological activity’ (Somjen, 1979).

## AUTHOR CONTRIBUTIONS

Contribution and design of the experiment: Angus M. Brown. Collection of data: Amy J. Hopper. Analysis and interpretation: Angus M. Brown and Amy J. Hopper. Drafting the article: Angus M. Brown and Amy J. Hopper. Both authors have read and approved the final version of this manuscript and agree to be accountable for all aspects of the work in ensuring that questions related to the accuracy or integrity of any part of the work are appropriately investigated and resolved. All persons designated as authors qualify for authorship, and all those who qualify for authorship are listed.

## CONFLICT OF INTEREST

None declared.

## Supporting information



Supplementary Material.

Supplementary Material.

## Data Availability

The data are available on request from the corresponding author.
